# Notes and News

**Published:** 1894-06-23

**Authors:** 


### Notes and News.

Collected and Communicated.

It is proposed to enlarge the United States Naval Hospital at Brooklyn, and to make it one of the finest naval hospitals in the world. In the meanwhile there is a dearth of candidates for the Naval Medical Corps, which would point to the fact that posts therein do not hold out great attractions at present.

A most suitable memorial is proposed in memory of the late Dr. Jolly, of Birmingham, to take the form of the endowment of a bed at the General Hospital, the scene of his labours and the centre of his professional interest. Dr. Jolly himself in his lifetime was a generous donor of the institution.

A new French hospital is being built at Constantinople, the corner stone of which has already been laid. The projected building, we hear, is to be an exceptionally handsome and well-appointed institution. An old infirmary in one of the fine squares of Pera—a suburb of the Turkish Capital—has given room for the French hospital.

We endeavour through the medium of our columns to enable our readers scattered all over the world to keep touch with the institutions they are interested in, and acquaint them with the work done and progress made. For this purpose we are greatly dependent on the annual reports of hospitals kindly sent us. Facilities afforded us by the compilers of the reports naturally vary in completeness, but we do not recollect having met with a record so hopelessly inadequate as the annual report of the Bedford Infirmary. The arrangement of the matter is confusing. The facts are given in tabulated form of an antique order. The accounts naturally, therefore, are not given under the uniform system, and as to a statement of the work of the year there is absolutely none. A great deal of interest is evinced in the infirmary by the people of Bedford, and matters relating to it are well aired in the local press. We can hardly imagine that the governors of the institution can much longer rest content with the uniquely inadequate report of their infirmary—a report which is not calculated to arouse any interest in, or add to the credit of, a really excellent institution.

The terrible plague which has raged for some time in Hong Kong is still claiming numbers of victims. There is a diminution in the daily number of deaths, owing, it is said, to the flight of the terror-stricken population to Canton. Canton was the original seat of the disease, and in March and April as many as 500 deaths were reported in one day. The Chinese are strongly averse to sanitary measures, and show a great dread of entering hospitals. Our Army Medical Corps and the military have done their best to assist in the face of great opposition.

How do modern nerves weather the revelations of invisible dangers that are constantly being disclosed? The army of microbes reveals itself above, below, and around us in every direction. Railway carriages are discovered to be one of their favourite haunts through the investigation of the Russian Health Department. The Russian microbe crowds 78,000 of its kind into the square inch of dust on the floor of a third-class carriage. It is said that in Germany the microbe is equally sociable when travelling, and we in England are probably little less favoured. The idea is not a pleasant one, and if generally accepted as a fact, a popular travelling companion, consisting of a box of sanitas powder and a carbolic spray, should have ready sale.

The uses to which electricity may be turned are constantly revealing themselves. At the Royal Society Conversazione Mr. Snedekor exhibited the last tribute of electricity in aid of medical treatment. This exhibit consisted of an electric heat generator by which means a poultice could be kept to any heat desired whilst worn by the patient, an obvious advantage, dispensing with the constant changing of fermentation, often necessary where a high temperature has to be maintained. The same method could be applied to hot-water bottles, obviating any inconvenience in refilling, and for various other purposes. The invention is ingenious and likely to prove serviceable, and we hope to give a technical description and illustration of it in our columns at a later date.

Amongst the men who have made modern psychiatry in this country in addition to those already mentioned by Dr. Clouston in the series of articles now appearing in our columns, we must not omit to include the following: Batty Tuke, by his pathological works and by his "open door system," has done his part of the work. Men who have done such good work as Coxe, Sibbald, Fraser, Lawson, Ley, Hitchman, Holland, Yellowlees, Robertson, Campbell, Rutherford, Rorie, Newington, and Howden well deserve a place in any notice of those who have made modern psychiatry. Howden's clinical and pathological work and his "Pathological Index" have added to our resources against disease. The veteran and fearless Mould, of Cheadle, has taught us all that an institution for the better class insane may be scattered over four counties. In the department of idiocy and congenital mental defects, Langdon Down, Ireland, Beach and Shuttleworth, by their clinical work and contributions to literature, as well as by their administrative work, have well kept up the reputation of this country. In criminal anthropology and psychology we have not lately kept our ground, though Thompson and Wilson began well, and Orange and Nicolson have done good work both by their writings and at Broadmoor Criminal Asylum. If we have not produced fascinating books on the subject we have practically solved the problem of providing for the criminal insane.

